# Methods for Indirect Treatment Comparison: Results from a Systematic Literature Review

**DOI:** 10.3390/jmahp12020006

**Published:** 2024-04-16

**Authors:** Bérengère Macabeo, Arthur Quenéchdu, Samuel Aballéa, Clément François, Laurent Boyer, Philippe Laramée

**Affiliations:** 1 Department of Public Health, Aix-Marseille University, 13005 Marseille, France; 2 Pierre Fabre Laboratories, 92100 Paris, France; 3 Amaris, Montréal, QC H2Y 2N1, Canada; 4 InovIntell, 3023GJ Rotterdam, The Netherlands

**Keywords:** systematic literature review (SLR), indirect treatment comparison (ITC), oncology, network meta-analysis (NMA), Bucher, matching-adjusted indirect comparison (MAIC), methodology, methods

## Abstract

Introduction: Health technology assessment (HTA) agencies express a clear preference for randomized controlled trials when assessing the comparative efficacy of two or more treatments. However, an indirect treatment comparison (ITC) is often necessary where a direct comparison is unavailable or, in some cases, not possible. Numerous ITC techniques are described in the literature. A systematic literature review (SLR) was conducted to identify all the relevant literature on existing ITC techniques, provide a comprehensive description of each technique and evaluate their strengths and limitations from an HTA perspective in order to develop guidance on the most appropriate method to use in different scenarios. Methods: Electronic database searches of Embase and PubMed, as well as grey literature searches, were conducted on 15 November 2021. Eligible articles were peer-reviewed papers that specifically described the methods used for different ITC techniques and were written in English. The review was performed in accordance with the Preferred Reporting Items for Systematic Reviews and Meta-Analyses (PRISMA) guidelines. Results: A total of 73 articles were included in the SLR, reporting on seven different ITC techniques. All reported techniques were forms of adjusted ITC. Network meta-analysis (NMA) was the most frequently described technique (in 79.5% of the included articles), followed by matching-adjusted indirect comparison (MAIC) (30.1%), network meta-regression (24.7%), the Bucher method (23.3%), simulated treatment comparison (STC) (21.9%), propensity score matching (4.1%) and inverse probability of treatment weighting (4.1%). The appropriate choice of ITC technique is critical and should be based on the feasibility of a connected network, the evidence of heterogeneity between and within studies, the overall number of relevant studies and the availability of individual patient-level data (IPD). MAIC and STC were found to be common techniques in the case of single-arm studies, which are increasingly being conducted in oncology and rare diseases, whilst the Bucher method and NMA provide suitable options where no IPD is available. Conclusion: ITCs can provide alternative evidence where direct comparative evidence may be missing. ITCs are currently considered by HTA agencies on a case-by-case basis; however, their acceptability remains low. Clearer international consensus and guidance on the methods to use for different ITC techniques is needed to improve the quality of ITCs submitted to HTA agencies. ITC techniques continue to evolve quickly, and more efficient techniques may become available in the future.

## 1. Introduction

### Background and Rationale

When assessing the comparative efficacy of new treatments, health technology assessment (HTA) agencies express a clear preference for randomized controlled trials (RCTs) as the gold standard for presenting evidence of clinical efficacy and safety [1,2]. RCTs allow a direct head-to-head comparison between two or more interventions and are, therefore, considered to be the most reliable source of comparative clinical efficacy and safety evidence [3].

However, in many situations, it can be unethical, unfeasible or impractical for new treatments to be compared directly to the most appropriate comparator through an RCT. In some cases, RCTs may compare the intervention of interest against placebo rather than the most appropriate comparator, which may vary by country and change over time with the availability of new evidence. Ethical considerations may also make a direct comparison impossible, as is often the case when developing treatments for life-threatening diseases. Moreover, direct comparison can be infeasible, such as for rare diseases where patient numbers can be very low [4,5,6]. Finally, if multiple comparators are of relevance to a particular indication, an RCT directly comparing all comparators of interest may be impractical.

While indirect treatment comparisons (ITCs) do not replace RCTs, they can provide useful evidence for aiding HTA agencies with decision making in cases where no direct comparison is available. Additionally, in situations where appropriate RCTs are available, ITCs can provide complementary evidence that can also be of use to inform decision making. Numerous ITC techniques exist in the literature, and these are continuing to evolve quickly. Naïve comparisons, whereby study arms from different trials are compared as if they were from the same RCT, are generally avoided due to their susceptibility to bias; the effect of a treatment may be over- or under-estimated, and, therefore, ITC techniques allowing for an adjusted indirect comparison are preferred. Given the variation in methodology underpinning different ITC techniques and the increasing importance of ITCs in providing evidence for therapies in HTA evaluations, the need for a systematic literature review (SLR) of possible ITC techniques detailed in the published literature was identified.

This SLR was conducted to identify the relevant literature on existing ITC techniques, provide a comprehensive description of each technique and evaluate their strengths and limitations from an HTA perspective in order to develop guidance on the most appropriate method to use in different scenarios.

## 2. Methods

### 2.1. Search Strategy and Selection Criteria

An SLR was conducted in accordance with the Preferred Reporting Items for Systematic Reviews and Meta-Analyses (PRISMA) guidelines [7]. Systematic searches were conducted in the Embase and PubMed electronic databases for a period from database inception to 15 November 2021, and manual hand searches were conducted to identify relevant documents across the following HTA and regulatory agency websites: National Institute for Health and Care Excellence (NICE); Haute Autorité de Santé (HAS); Institute for Quality and Efficiency in Health Care (IQWiG); European Network for Health Technology Assessment (EUnetHTA); Canadian Agency for Drugs and Technologies in Health (CADTH); Pharmaceutical Benefits Advisory Committee (PBAC); US Food and Drug Administration (FDA); the European Medicines Agency (EMA); and Therapeutic Goods Administration (TGA).

The full search strategy is provided in Supplementary Data, Tables S1 and S2. Initial searches using key words relating to ITC techniques alone resulted in an excess of hits and, as such, an alternative approach was taken. In addition to key words based on ITC techniques, the revised search strategy included key words based on journal name and author name from a predefined list of methodological articles of ITCs. This list was developed from methodologically well-known and referenced peer-reviewed papers and grey methodological literature/reports (such as the technical support documents [TSDs] produced by the NICE Decision Support Unit [DSU]), recent publications, targeted searches of the peer-reviewed and grey literature, and reviews of reference lists of the articles identified (Supplementary Data, Table S3). 

All articles identified through the searches were imported to Microsoft Excel; primary-level screening of titles and abstracts was conducted against predefined eligibility criteria. Duplicates were removed to evaluate the studies for full-text eligibility. Eligible studies included those containing information on the methods of ITC techniques that were written in English, while studies reporting the application of ITC techniques solely in the context of specific treatments were excluded. The SLR eligibility criteria based on the PICO (Population, Intervention, Comparator[s], Outcome[s]) framework is presented in Supplementary Data, Table S4). However, since the objective of this SLR was not to investigate specific treatments or diseases, the PICO framework was not considered sufficient, and additional inclusion and exclusion criteria were utilized (Table 1).

### 2.2. Data Extraction and Synthesis

Primary-level screening of titles and abstracts against the eligibility criteria in Table 1 was first conducted, followed by full-text review. Both of these steps were completed by two independent reviewers, and at each stage, any discrepancies were discussed and resolved by a third party.

A data extraction table was created in Microsoft Excel to assimilate data from all eligible articles, from which the data were then extracted. The extracted data included the study design (including study type, objectives, endpoints, and individual patient data [IPD] availability), ITC technique evaluated, methods described for ITC technique(s), strengths and limitations, and further methodological considerations for each ITC technique. Data extraction from the included articles was conducted by one reviewer, with the extracted data independently checked by a second reviewer; any disparities were referred to a third party. In order to conduct a qualitative review of the methods underpinning each ITC technique, extracted information was then collated and analyzed by ITC technique.

## 3. Results

### 3.1. Identification of Articles

**Figure 1:**
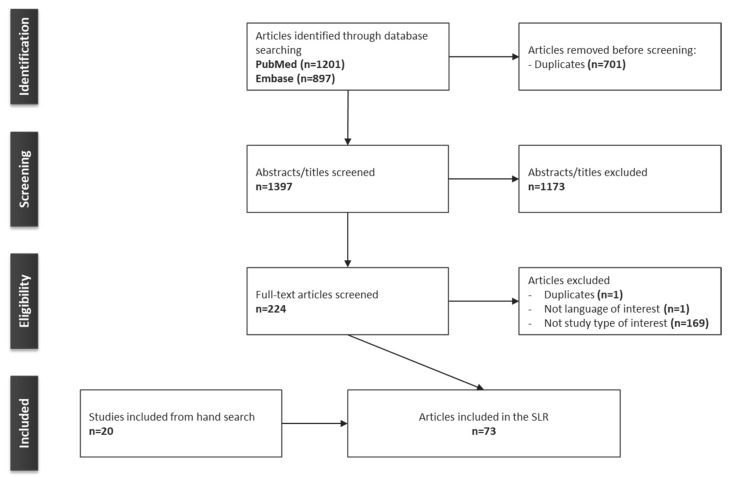
PRISMA flow diagram of the SLR. **Abbreviations:** PRISMA, Preferred Reporting Items for Systematic Reviews and Meta-Analyses; SLR, systematic literature review.

The electronic database searches identified a total of 2098 articles (PubMed searches yielded 1201 results; Embase searches yielded 897 results). After the removal of 701 duplicates, 1397 articles were screened by title and abstract. At this stage, 1173 articles were excluded, leaving 224 potentially relevant articles. The full publications for these were then screened, resulting in the exclusion of a further 171 articles based on the following criteria: not a study type of interest (n = 169), not a language of interest (n = 1), and duplicates (n = 1); 53 articles were, therefore, eligible for inclusion in the SLR. Hand searches were also conducted, from which 20 articles were included, resulting in a total of 73 articles included in this review (Figure 1).

### 3.2. Description of the Included Articles

**Figure 2:**
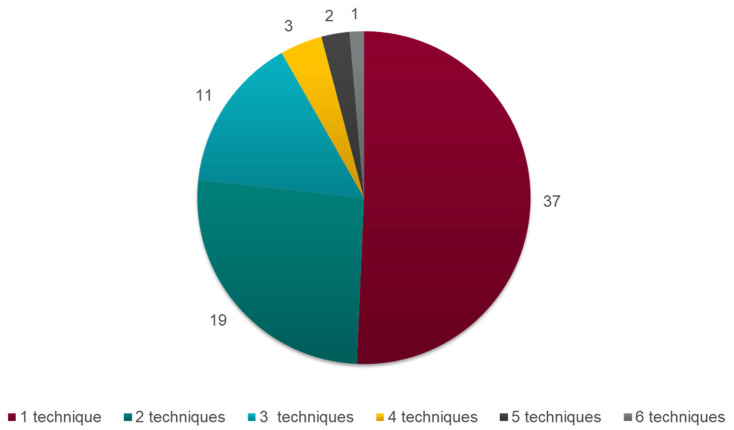
Pie chart of the number of ITC techniques described per article included in the review. **Abbreviations:** ITC, indirect treatment comparison.

Among the 73 included articles, seven different ITC techniques were reported. Network meta-analysis (NMA) was the most frequent technique described (in 79.5% of the articles), followed by matching-adjusted indirect comparison (MAIC; 30.1%), network meta-regression (NMR; 24.7%), the Bucher method (23.3%), simulated treatment comparison (STC; 21.9%), propensity score matching (PSM; 4.1%), and inverse probability of treatment weighting (IPTW; 4.1%). Among recent articles (published from 2020 onwards), the majority describe population-adjusted methods, notably MAIC (9/13; 69.2%). More than half of the articles described only one technique (37 articles; 50.7%), and six articles included a description of four or more techniques (Figure 2).

In addition to the ITC techniques themselves, several methodological considerations were described in the included articles. In terms of statistical approach with regard to NMAs, at least one of either ‘frequentist’ or ‘Bayesian’ methods were explicitly mentioned in 32 (44%) articles. Fixed-effects and random-effects modeling were also described in the literature, with at least one of these two frameworks being described or used in 28 (38%) of the included articles. The notion of ranking was mentioned in 12 articles (16%), and concerns over inconsistency and heterogeneity were included in 23 (32%) and 26 (36%) articles, respectively. Other concepts, such as imprecision, incoherence, dealing with missing data and validation of results, were also described in a minority of the included articles.

### 3.3. Summary of the Methods for ITC Techniques

The ITC techniques identified in the SLR all utilized methods of adjusted indirect comparison. These methods are either based on a connected network and estimate relative treatment effects via a shared comparator, therefore respecting the randomization of included clinical trials, or they are population-adjusted methods, which seek to ensure comparability between populations through adjustment based on treatment effect modifiers (TEMs). In contrast, unadjusted ITCs (such as naïve ITCs) negate the randomized nature of each individual RCT by comparing absolute outcomes, and, therefore, adjusted ITCs should always be used in the first instance [8,9].

Among adjusted ITCs, the standard ITC techniques such as the Bucher method and NMA assume that there are no cross-trial differences in the distribution of effect-modifying variables (more specifically, that relative treatment effects are constant), therefore producing biased estimates when cross-trial differences exist. To undertake an ITC, basic assumptions are required (Table 2). The most fundamental assumption is exchangeability, which is tested by assessing the properties of homogeneity (as per standard meta-analysis), similarity and consistency [2,8,9,10,11,12,13]. To provide a robust relative treatment effect estimate, studies included in the ITC should be sufficiently similar in terms of study design, patient characteristics, treatments and outcomes measured [13]. When there are cross-trial differences in effect modifiers, implying that relative treatment effects are not constant across trial populations, several methods known as population-adjusted indirect comparisons have been introduced to estimate relative treatment effects [14].

A lack of consensus regarding the terminology associated with ITCs was identified in the literature [12]. The terminology adopted for the assumptions and other definitions in this review are described in Table 2.

Before initiating an ITC, an SLR should be performed to identify all available studies of interest for consideration [3,8,15]. A feasibility assessment should then be conducted to retain only the studies that are sufficiently similar in terms of study design, patient characteristics, treatments and outcomes measured (in terms of both data availability and definitions of endpoints) for inclusion in the ITC. Consideration should be given to the types of outcomes and corresponding measures of interest compared through ITCs, which can include binary outcomes (relative risk [RR], odds ratio [OR], risk difference [RD]), continuous outcomes (mean difference, standardized effect size), count data and time-to-event (TTE) outcomes (rate ratios, hazard ratios [HR]). The results of the feasibility assessment will then inform the choice of ITC technique that can be utilized [3,8,15].

**Figure 3:**
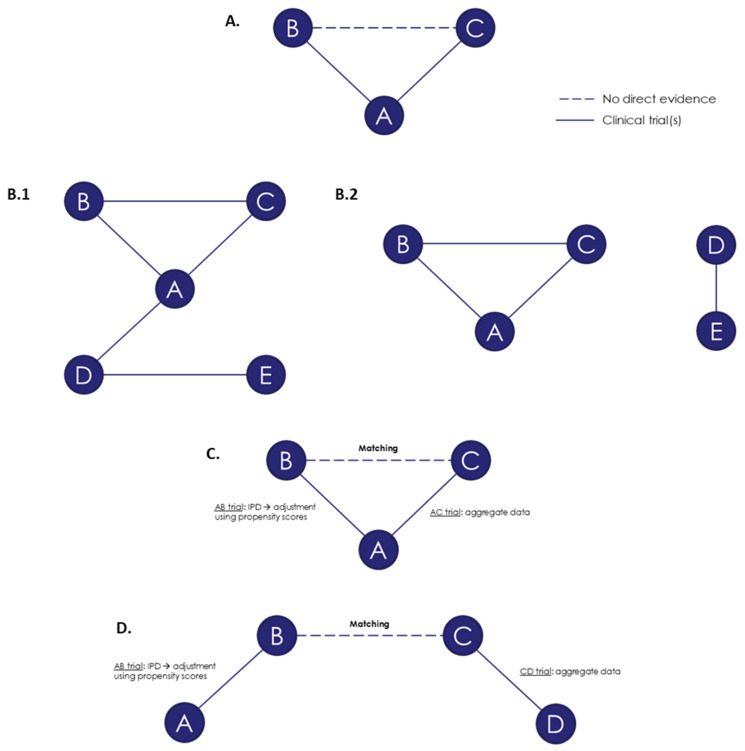
A, B, C, D and E are treatments. (**A**) network of evidence for an ITC using the Bucher method; (**B**) NMA examples of connected (**B.1**) and disconnected (**B.2**) networks; (**C**) anchored MAIC; (**D**) unanchored MAIC. Abbreviations: IPD, individual patient data; ITC, indirect treatment comparison; MAIC, matching-adjusted indirect comparison; NMA, network meta-analysis.

The ITC techniques identified in the literature from this SLR are summarized in Table 3 and Figure 3, and are described in more detail in the below sections.

#### 3.3.1. The Bucher Method for Adjusted ITC

The earliest and simplest technique for ITC was introduced by Bucher et al. in 1997 [16]. The Bucher method is an adjusted ITC technique for aggregate data (AgD) that can estimate relative treatment efficacy in a simple network of three different treatments (A, B and C) where no direct evidence exists between the two treatments of interest (e.g., B and C [Figure 3A]) [16,17]. The evidence is, therefore, indirect through comparison with the common comparator, treatment A [8,13,14,15,16,17,18,19,20,21,22]. In these situations, the consistency assumption cannot be assessed, and a thorough assessment of homogeneity and similarity is particularly important [15,17,21]. The Bucher method can also be applied in situations of a “single loop of evidence” where there is direct evidence in the form of an RCT comparing B and C; however, this approach is less frequently used.

This technique is adequate when there is only one study per pairwise comparison, but for cases in which there are multiple studies for comparison, these must first be combined to obtain a summary effect estimate. This requires the use of a traditional pairwise meta-analysis approach. The Bucher method uses ORs as a measure of treatment effect, although it can be extended to utilize other measures such as RR, RD, standardized mean difference and HR [17].

The first step is to independently synthesize the evidence in each pairwise comparison [14]. The direct estimates of the effects of B versus A and A versus C are then combined to obtain the relative treatment effect of B versus C by measuring the difference between the treatment effects of AB and AC. The variance of the effect of BC is the sum of the variances of the effect of AB and AC [15].

The Bucher method assumes independence between the pairwise comparisons; therefore, the method cannot readily be applied to multi-arm trials where all trial interventions are taken into account within the same comparative analysis [20]. This technique also relies on the assumption of homogeneity and similarity between the AB and AC trials. As such, homogeneity and similarity must be assessed to ensure that there are no differences in the distribution of TEMs; see definition in Table 2) [20].

A key strength of the Bucher method is that the effect measure comparing two treatments from an RCT is used as opposed to the individual results for each of the treatment groups, ensuring that the strength of randomization is preserved and study-level differences in prognostic factors do not bias results [18]. For cases of multiple studies for a pairwise comparison, the effect measures for each RCT comparing A to B are combined and then compared to the corresponding combined effect measure based on the RCTs comparing A to C. This is in contrast to naïve ITCs, which compare study arms from different trials as if they were from the same RCT, despite the randomization linking treatment groups being broken [14,20].

However, since the Bucher method does not adjust for TEMs, results may be biased if the TEMs are imbalanced. This technique is also limited to a simple network of only three treatments and cannot be used for multi-arm trials taking into account all the trial interventions in the same comparative analysis, with the pairwise comparison estimates being correlated [14,20,22].

#### 3.3.2. NMA

A traditional pairwise meta-analysis involves comparing two treatments by pooling the pairwise comparisons of several RCTs that have evaluated these two treatments. An NMA, also known as a mixed treatment comparison (MTC), extends the technique of the traditional pairwise comparison by comparing multiple treatments simultaneously in a single analysis [2,8,23]. The results of multiple studies are combined to estimate the relative treatment effect, taking account of direct and/or indirect evidence. Where evidence is available, NMAs should include all available relevant comparators [2,8,12,13,21,23,24,25,26,27,28,29,30,31,32].

The first step of an NMA involves conducting an SLR to identify all available studies of interest, followed by a feasibility assessment to exclude any studies that differ significantly in terms of characteristics that may impact the treatment effect. Networks of evidence based on connections between studies via their comparators are built per outcome, and the network must be connected in the manner shown in Figure 3B in order for the NMA to be conducted. The validity of common comparators must then be assessed by comparing baseline characteristics and study designs. Homogeneity, similarity and consistency should also be explored, as both heterogeneity and inconsistency can arise due to the presence of TEMs and, therefore, as a result of differences in trial populations, study design, setting and length of follow-up. Firstly, to assess clinical heterogeneity, comparisons of baseline characteristics across the studies, as well as trial design, eligibility criteria and outcome definitions should be conducted. Potential TEMs must be identified based on clinical studies and clinical expert opinion. Statistical heterogeneity may then also be explored at the analysis stage, at which point descriptive statistics on TEMs and a statistical test for direct comparison should be conducted. Measures of inconsistency can be carried out for NMAs, for which both direct and indirect evidence is available. A statistically significant difference in the estimates of relative effectiveness between direct and indirect evidence would indicate inconsistency [8]. Checks of inconsistency should be carried out using established methods such as a node-splitting approach upon completion of the NMA.

If either heterogeneity or inconsistency is identified, several approaches can be considered, including sensitivity analyses that exclude outlier studies from the networks, subgroup analyses, exclusion of trials or NMR (see NMR) [25,26]. Then, if the NMA is considered feasible, the analysis can be conducted for each endpoint of interest using either a random-effects (preferable when heterogeneity is identified) or fixed-effects model (see Additional Statistical Considerations), and a Bayesian (often using the statistical software WinBUGS^®^ http://www.bayesianscientific.org/resource/bugs-openbugs-winbugs/ accessed on 18 July 2023) or frequentist framework (see Additional Statistical Considerations) [31].

As the last step, the convergence and validity of the results should be evaluated. To evaluate internal validity, the number of studies and quality of included RCTs should be considered. External validity should be evaluated by considering any adjustments that have been made to assess heterogeneity.

NMAs rely on the assumption of connectivity within the network, as well as homogeneity, similarity and consistency between trials. NMAs also assume that there is constancy of relative effects (as such there should be no differences between trials in the distribution of TEMs), which is one of the key limitations of the technique and is discussed further below [31].

Similarly to the Bucher method, NMAs rely on a connected network of RCTs, thus, the strength of randomization is preserved and differences in prognostic factors across studies do not bias the results. Another key advantage of NMAs is that the number of trials that can be included is unlimited. In the event that more than one comparator is used to perform an ITC, NMAs can incorporate each comparator into a single model through pathways and different routes between indirect comparators to arrive at an indirect estimate of treatment effect. The level of agreement in the results obtained from the different pathways for the indirect comparison can then be quantified. Furthermore, Lumley et al. (2002) proposed that the combination of both direct and indirect evidence may result in a narrower confidence interval around the treatment effect than would be obtained if the relative efficacy of two treatments was based on limited direct evidence alone. However, the statistical power and precision of the indirect comparisons made with NMAs are dependent on the number of trials, sample size and statistical information available [33,34,35]. Finally, NMAs can be conducted using IPD instead of AgD, which can allow for more flexibility in the analysis [27].

A key limitation of NMAs is that they assume conditional constancy of relative effects, where relative treatment effects are constant between populations at any particular level of a set of covariates, and the correlation between covariates is ignored [8]. This technique does not adjust for the bias introduced by an imbalance in TEMs between studies, which may lead to inconsistencies across the network. In such cases, an NMR can be conducted instead. Moreover, NMAs do not account for correlations that may exist between different effect estimates when they are obtained from a single multi-arm trial. Although a random-effects model can be used, in which multi-arm correction is applied, this is not considered to be an optimal solution [17,36]. The use of a Bayesian approach to appropriately model random-effects in multi-arm trials has also been questioned due to its complexity and subsequent concern over the sensibility of conclusions drawn.

Another limitation of NMAs is that different pathways may involve overlap, referring to the situation where two or more studies in the network share at least one common treatment arm; when the level of inconsistency is estimated, the NMA method does not account for any overlap that may exist. Given overlap cannot be accounted for, in situations where the same comparison is performed via different pathways, the estimated inconsistency will be less than the true amount [26,30]. This is because the true inconsistency between the result of a comparison and the result of the same comparison via a different pathway should be zero since the same data set is used each time. Finally, substantial trial power reduction strongly reduces the likelihood of demonstrating superiority between interventions when trials are introduced into an NMA [35].

### 3.4. Population-Adjusted Methods for Indirect Comparisons

Unlike NMA and the Bucher method, population-adjusted indirect comparison methods seek to adjust for imbalances in TEMs using IPD from one or more studies [37]. The assumption of similarity is relaxed and the main assumption is conditional constancy of effects (relative or absolute). The population-adjusted methods for indirect comparison described in this SLR were MAIC, STC, NMR and propensity-score based techniques. For all of these techniques, the first step in conducting the analysis involves the identification of potential TEMs.

#### 3.4.1. MAIC

MAIC is a population-adjusted method specifically designed for a two-study indirect comparison scenario, applying propensity score weighting (PSW) (see PS-Based Techniques). It requires IPD from at least one trial (e.g., the AB trial in Figure 3C) because the aim is to match the IPD to the AgD of the comparator arm from another trial (e.g., the AC trial), for which IPD are unavailable [8,18,37,38,39,40,41,42,43,44,45].

To conduct a MAIC, potential TEMs must be chosen carefully based on the literature, clinical expert opinion and descriptive analyses of IPD. The inclusion criteria of the AgD trial (AC trial) are then applied to the trial with IPD (AB trial); therefore, some patients may be excluded before conducting the analysis at this stage. The population of the IPD trial should also then be adjusted to match the AgD population, and PS should be generated for all patients by adjusting based on the identified potential TEMs and re-weighting the patients in the IPD trial to match the covariate distributions of the AgD trial. Re-weighting ensures that the IPD and AgD populations are matched in terms of confounding covariates [44]. PS are commonly generated using logistic regression, but other methods such as machine learning can also be used. PS are defined as the conditional probability for an individual to receive the treatment given their initial characteristics (i.e., prespecified confounders including individual baseline demographic factors and prognostic factors). The mechanism for adjustment is a logistic regression model to predict weights related to individual patients for each comparator of interest with available IPD. To make the adjustment, there needs to be overlap in the distributions of the covariates in each study. Individual weights are then defined using the inverses of PS, and adjustment is made on the entire population using these weights. A MAIC should not be performed if insufficient overlap is observed between the populations, even after adjustment [37].

MAICs compare the outcome of the patients with IPD calculated with re-weighting with the AgD to obtain the relative effect [45]. Effective sample size (ESS) is used to give an indication of the amount of information retained in the trial after re-weighting.

MAICs can be either anchored (Figure 3C), where there is a common comparator creating a connected network, or unanchored (Figure 3D), in the absence of a common comparator. An unanchored network is a disconnected network, using data from RCTs or single-arm trials [45].

Unanchored MAICs require the assumption that all prognostic variables and TEMs have been included in the adjustment model (there are no unobserved prognostic variables and TEMs), while anchored analyses only require the assumption that all TEMs have been included. Due to the common comparator, anchored MAIC estimates are theoretically not biased by the existence of unbalanced prognostic variables that are not TEMs. For pairwise comparisons where TEMs are unbalanced across studies, MAICs incorporate adjustment for TEMs, whereas Bucher and NMA methods do not. This double adjustment (on the common comparator, with population adjustment on the TEM) makes MAICs a more complex and time-consuming method overall than NMA. There is also uncertainty regarding the distribution of study-level differences, both measured and unmeasured, that may influence the outcome of interest due to the isolation of treatment arms in the analysis. In cases where IPD are available, this additional information may allow for a more selective population to be matched to external studies in terms of between-study differences in patient characteristics, such as treatment experience. Unanchored MAICs should be expected to have greater uncertainty than anchored analyses, and may be applied as a method of reconnecting a network to facilitate an NMA as a second step for treatments that were not originally compared, even indirectly [10].

To phrase this differently, anchored MAICs assume conditional constancy of relative effects, meaning that all relevant TEMs are included in the model. Unanchored MAICs make the stronger assumption of conditional constancy of absolute effects, meaning that the absolute treatment effects are constant across all TEMs and prognostic variables, and all of these factors are known. This latter assumption is considered near impossible to meet [10].

#### 3.4.2. STC

STC is a population-adjusted method specifically designed for an indirect comparison between two studies based on outcome regression methods [14,18,33,37,41,42,43,44,46]. They are similar to MAICs in that they generate adjusted responses for a treatment in a study for which there are IPD in order to match the baseline characteristics of patients who received a comparator of interest in another study, but differ in the way that the adjustments are made [33,37].

The initial steps of an STC are similar to those described above for MAICs. It is again more efficient to use a reduced rather than maximum number of covariates for adjustment. Although the aim is to adjust for all TEMs, as with MAICs, it is more computationally efficient to adjust for a select rather than maximum number of covariates. The STC method uses predictive equations such as generalized linear models to estimate the relationship between the outcome and baseline characteristics. The inclusion criteria of the AgD trial (AC trial; see Figure 3C) should then be applied to the one with IPD (AB trial); the population of the IPD trial can then be adjusted to match the AgD population. The mechanism for adjustment is an equation of regression methods for each outcome of interest; an outcome regression model is fitted using the IPD baseline characteristics and treatment from the AB trial (Figure 3C) to predict the average effect of A versus B in the AC population, dependent on the covariates of the AC AgD trial, and, finally, a population-adjusted average effect of B versus C in the AC population [8,33]. As effect-modifying covariates are likely to be good predictors of outcome, the inclusion of appropriate TEMs should provide an acceptable fit. Similarly to MAICs, STCs can be either anchored (Figure 3C), where there is a common comparator creating a connected network, or unanchored (Figure 3D) in the absence of a common comparator [37].

Unanchored STCs assume that there are no unobserved prognostic variables or TEMs. It is also assumed that there is some overlap between the distributions of the selected covariates in AB and AC. This assumption does not hold if the eligibility criteria of AC and AB are inconsistent. Anchored STCs also hold the additional assumption of conditional constancy of relative effects, while unanchored STCs hold the assumption of conditional constancy of absolute effects.

#### 3.4.3. Comparison of MAIC and STC

For pairwise comparisons where TEMs are unbalanced across studies, MAICs and STCs incorporate adjustment for TEMs, whereas the Bucher and NMA methods do not. MAICs and STCs can be used in cases where the evidence network is incomplete, allowing for the comparison of treatments across studies in which there is no common comparator, as well as the inclusion of single-arm studies. Unanchored MAICs/STCs require both prognostic factors and TEMs to be specified, while anchored MAICs/STCs only require the specification of TEMs. MAICs and STCs can also be particularly useful in situations of heterogeneity, where the selected trials form a connected network, but the effect of the treatment assessed in the studies is altered by one or more interaction variables, and the distribution of these variables differs among the various selected trials. The reliability of NMAs in this situation may be compromised as heterogeneity can impact comparisons at intermediate steps (branches in the network) and distort the main comparison of interest, leading to substantial uncertainty in estimating the relative effects [8]. The targeted comparisons involved in STCs and MAICs bypass the issue by targeting the analyses on specific arms of interest, as long as the trials of treatments A and B can be considered sufficiently compatible for a targeted comparison [8].

MAICs and STCs are limited to pairwise indirect comparisons and can be extended to larger networks only by repeating the pairwise analysis for all pairwise comparisons of interest. MAICs and STCs are problematic when there are more than two studies, as population adjustment differs for each study to which they are being adjusted. MAICs and STCs are also limited in that they are only able to estimate treatment effects in the AgD trial population, which may not match the target population for the decision. Therefore, if MAICs were conducted for treatments B and C based on the same two trials but using separate IPD for each treatment, the results would be relevant for two different populations, each likely to have substantial differences due to the differences in population across studies. Furthermore, the target population for the decision may be a different population again, which could lead to alternative and potentially spurious recommendations.

Another limitation is that MAICs and STCs carry numerous risks of bias. Results are biased if not all relevant TEMs have been adjusted for (in addition to prognostic variables in unanchored settings); adjusting for covariates that are not TEMs or for irrelevant factors reduces the precision of the estimates without increasing the reliability of the estimates. MAICs and STCs only account for differences in patient populations between trials via the inclusion of covariates in the adjustment model. However, they do not account for differences in study design, such as the phase of study. As such, any potential biases due to study design should be described. In addition, both MAICs and STCs combine an adjusted treatment effect estimate with an unadjusted estimate from the AgD study. Anchored MAICs and STCs rely on the assumption of conditional constancy of relative effects, where relative treatment effects are constant between populations at any particular level of a set of covariates. MAICs also cannot be conducted if the two study populations do not overlap at all, as this leads to large reductions in ESS and imprecise estimates of the treatment effect. There must also be sufficient patient numbers in the trial with IPD to confirm the summary outcomes [37]. The exclusion of balanced covariates does not ensure their balance after the weighting procedure, as including too many covariates or poor overlap in the covariate distributions can induce extreme weights and large reductions in ESS.

In contrast to STCs, MAICs require TEMs to be specified to the appropriate scale and measure in the model for the weights in order to achieve balanced TEMs after weighting. In the case of categorical outcomes, it would not be possible to adjust for a factor if a particular category is not represented in one of the studies. For continuous outcome measures, it may not be possible to weight the values for which there are IPD so that they match the average baseline value in the comparator decision set. Extreme weights arise when there is poor overlap in the joint distribution of covariates between studies. Unlike traditional PSW the availability of AgD for some trials in a MAIC prevents the use of existing methods for checking the fit and calibration of the PS model.

Despite similarities between these methods, STCs have some additional limitations in comparison to MAICs. The relative precision and accuracy of STCs deteriorates if the terms corresponding to the prognostic covariates are not included in the outcome regression or the incorrect omission of TEMs leads to inaccurate specification and poor precision. STC results may be strongly influenced by the assumed relationship between a covariate and an outcome; a linear relationship is often assumed. Both MAIC and STC methods can incorporate TTE data, but a parametric distribution must be specified in STCs. However, one advantage of STCs is that, given that they can extrapolate beyond the range of the IPD using the linearity assumption or other appropriate assumptions, they can produce estimates even when there is no overlap between study populations.

From a practical perspective, STCs are more suitable when there is an interest in multiple comparators for few outcomes. However, there are situations in which a MAIC may be more appropriate than an STC, such as if the analysis is intended to be repeated for only a few comparators but multiple outcomes are to be studied [43]. MAICs also offer flexibility for the analyses of TTE outcomes and those requiring non-linear models (such as logistic), as well as in situations where the predictive equations derived for STC offer poor fit [40].

#### 3.4.4. NMR

NMR and multi-level NMR (ML-NMR) are regression-adjustment methods that incorporate trial-level covariates to not only accommodate but also explain between-study variability and adjust for heterogeneity between trials [8,17,38,39,46,47]. Covariates included in the regression model are usually TEMs selected based on clinical expert opinion. NMRs are an extension of the NMA framework, whereby, in the absence of TEMs, NMRs are equivalent to a standard NMA and would produce the exact same results. NMRs may incorporate all available data, including only AgD (NMR), only IPD (IPD-NMR), or any mixture of IPD and AgD (ML-NMR). The NMR approach assumes common regression coefficients at both the individual and aggregate level, which leads to aggregation bias (a form of ecological bias, which can arise from interactions between variables that are characterized by different scales) when the model is non-linear, whereas the ML-NMR method avoids such aggregation bias. NMRs can also be conducted to adjust for differences in baseline characteristics, such as non-comparable comparator or placebo arms across trials.

Initial steps of NMRs are the same as those of NMAs; SLRs and feasibility assessments are used to assess homogeneity, similarity and consistency across the studies of interest before the NMA and meta-regression can then be conducted. For ML-NMRs, an individual-level regression model is first defined, and AgD are subsequently fitted by integrating this model over the covariate distribution in each AgD study population, thus avoiding aggregation bias in the aggregate level model [47].

Similarly to NMAs, NMRs assume connectivity of the network of evidence. This approach also assumes that all TEMs have been adjusted for, i.e., there are no unobserved prognostic variables or TEMs. As with NMAs, NMRs also rely on the assumption of conditional constancy of relative effects [8,47].

Unlike MAICs and STCs, ML-NMRs are not limited to pairwise comparisons and are applicable to treatment networks of any size, including larger networks with any number of treatments or with any mixture of IPD and AgD, enabling use of all available information. ML-NMRs are also able to account for covariate correlation structures. In ML-NMRs, the population-adjusted treatment effects can be estimated for any target population with sufficient information on the covariate distribution, not just the population of the trial for which only AgD are available, as is the case with MAICs and STCs. In contrast, with STCs and traditional meta-regression approaches, including NMR, the ML-NMR method avoids aggregation bias by ensuring that the aggregate level model is appropriately related to the individual level model [47]. Marginal (population-average) treatment effects (average effects at the population level of moving an entire population from untreated to treated) may also be estimated using this method. Another advantage of STCs and NMRs is that they are able to extrapolate beyond the range of the IPD, producing estimates even when there is no overlap between study populations. IPD-NMR is the “gold-standard” approach to adjusting for differences in TEMs. NMR methods are also readily replicable, contrary to MAIC, which can be performed in different ways.

However, NMRs are not always feasible since their feasibility depends on the number of trials linking treatments, trial size, the level of heterogeneity between trials and the collection of covariates in all included trials. An NMR requires the number of trials, rather than the number of patients, to exceed the number of baseline characteristics used for adjustment. ML-NMRs carry further limitations and cannot adjust for large numbers of baseline differences, and they may be subject to aggregation bias due to unobserved TEMs, such as in MAICs and STCs. Furthermore, ML-NMRs depend on the assumption of conditional constancy of relative effects and the correct choice of regression model. This approach also requires either full IPD for at least one study investigating each treatment in the network, a sufficient number of AgD studies for each treatment, invoking the “shared effect modifier” assumption, or otherwise specifying informative prior distributions for treatment–covariate interactions. In practice, these requirements may be difficult to satisfy. NMRs and ML-NMRs also cannot be applied to multinomial or TTE data, although research is being conducted and, in contrast to unanchored MAICs and STCs, cannot include single-arm trials since the network must be connected [8,47].

#### 3.4.5. PS-Based Techniques

The PS-based techniques most commonly described in the literature are PSM and PSW [33]. These techniques require access to IPD for each arm of the analysis. This is in contrast to MAIC, which also uses PS but for which data are not available for all treatment arms of interest. PS-based techniques typically use a control arm from an observational study or RCT and another arm for patients who have received the treatment of interest from an RCT, often with a smaller sample size. PS is defined as the conditional probability of an individual being treated with a certain treatment of interest based on their baseline characteristics. Compared with ad hoc randomization in RCTs, PS is a post hoc randomization technique used to mimic what happens in RCTs by balancing covariates at the “randomization” point, thus substantially reducing the selection bias in observational studies. The relevant confounders (prognostic factors and TEMs) are determined based on a selection approach (see MAICs described above) and are included in a logistic regression model to estimate a PS.

Both PSM and PSW rely on the assumption that patients in both groups are eligible for the treatment of interest (defined here as positivity). There must also be sufficient overlap of the data available, as measured by PS, between the populations receiving the treatment of interest; the distribution of patients among the different PS values must be similar, and the populations in the groups being compared must be sufficiently balanced after adjustment for confounding. The degree of overlap and balance can also be influenced by excluding patients on the basis of PS values without overlap. If sufficient overlap and balance can be achieved in this way, the final overlapping and balanced population of patients is ultimately the target population to whom the estimated effects apply. Therefore, whether this target population sufficiently represents the population selected for the original research question should be explored. PS-based matching (PSM) methods and PS-based weighting methods, such as IPTW, can be used to improve the balance of potential confounders between the two treatments.

#### PSM

PSM aims to create a matched sample of data, in which distributions of baseline covariates are similar between treated and untreated patients, to better estimate the average effect of treatment exposure by regrouping patients with similar PS values across the treatment and control groups [8,29,48]. Patients from the trial of interest are matched to the most similar patient(s) from the comparator trial according to their PS values. Several parameters must be defined, including the structure of the matching, with or without replacement; the method (e.g., optimal matching, which considers all previously made matches before making a match; or greedy matching, where a match is fixed once it has been made); and the order of selection (e.g., random, lowest to highest). The method typically requires the number of patients in one trial to be larger than the other to allow for good matching of patient characteristics. Discarding unmatched observations reduces the size of the population and the treatment effect precision.

A strength of this approach is that patients who are too different can be excluded if treatment arms do not completely overlap. PSM also provides less biased estimates when extreme PS values are obtained through the PS model. However, estimates of treatment effect through PSM will be biased when there are unobserved prognostic variables and TEMs (as this causes PS model misspecification), as well as when there is poor overlap in the distribution of observed prognostic variables and TEMs, resulting in extreme PS values. The case of 1:n matching also has the limitation that not all patients are included in the final analysis, even though some patients could still provide valuable information.

#### PSW

The objective of IPTW is to control the influence of patients by weighting their responses based on their PS values. Treated patients are given a weight of 1/PS, and control patients are given a weight of 1/(1-PS). This approach estimates the average treatment effect among the entire population. Another approach is the standardized mortality/morbidity ratio weighting (SMRW), in which treated patients are given a weight of 1, and control patients are given a weight of PS/(1-PS). The SMRW method reweights the control patients to be representative of the treated patients, which results in an estimate of the average treatment effect among the treated population [48].

Unlike matching, weighting preserves sample size and, hence, can offer increased precision when estimating treatment effects by maximizing the available amount of information [29,42]. However, this approach still has the same limitation of PSM, whereby estimates of treatment effect will be biased when there are unobserved prognostic variables and TEMs or when there is poor overlap between the distributions of observed prognostic variables and TEMs. Furthermore, patients who are too different cannot be excluded if treatment arms do not completely overlap, and some patients can have very high weights and become overly influential. Correction methods and trimming approaches exist to handle extreme weights.

### 3.5. Additional Statistical Considerations

#### 3.5.1. Fixed-Effects and Random-Effects Approaches

A fixed-effects model assumes that the same single treatment effect exists in each study that compares the same treatments, as well as that variability is exclusively due to random variation, which is commonly implausible [21,25,33,49]. This approach is generally not advised in the presence of significant study heterogeneity.

A random-effects model assumes a different underlying effect for each study that compares the same treatments and takes this into consideration as an additional source of variation by considering a normal distribution for each treatment effect. However, the validity of this assumption may be difficult to verify, especially when the number of studies is small. When the results of small studies are systematically different from those of the large ones, the normality assumption is not justified either.

The choice of either a fixed- or random-effects model must be based on the convergence (if only one model converges, it should be selected), the presence of study heterogeneity (random-effects models must be prioritized if there is heterogeneity), and the deviance information criterion (DIC), which is a statistical criterion estimating the quality of a statistical model.

#### 3.5.2. Frequentist Versus Bayesian Approach

The ITC techniques described are based on similarity and consistency assumptions that adhere to Bayesian or frequentist statistical approaches. A Bayesian framework assumes a prior knowledge of probability models, while the frequentist approach derives probability from the frequency of events occurring over a number of repeated trials.

The Bayesian approach combines a prior probability distribution with a likelihood based on the available data. This results in a posterior probability distribution from which relative effect estimates can be obtained. Results can, therefore, be interpreted in terms of probabilities, and expressed as a posterior distribution from the mean, median, standard deviation and 95% credibility interval (CrI) can be derived [17,33,49,50,51,52]. The ranking of a treatment can also be interpreted by either the proportion of iterations for the order in which treatments are ranked or the probability of one treatment to perform better than each comparator. Considerable uncertainty remains around the value of a between-study heterogeneity parameter by using a noninformative prior in a small or sparse network. A potential solution for this is to use more informative priors for between-study heterogeneity.

In contrast to the Bayesian approach, in a frequentist framework, the parameter of interest is fixed. A frequentist approach uses the sampling distribution as the basis of statistical inference that is proportional to the likelihood function. Uncertainty around heterogeneity is usually ignored in the calculation of CIs. Results are expressed as point estimates, standard errors, 95% CIs, and *p*-values. Unlike the Bayesian approach, there is generally no ranking of treatments in frequentist NMA, even though work has been conducted by Rücker et al. (2015) to make this possible [13,53].

A Bayesian approach might be preferred to a frequentist approach as, for example, it allows for incorporation into the random-effects model of between-study heterogeneity, including a prior distribution for it. The main differences between the Bayesian and frequentist approaches are summarized in Table 4.

### 3.6. Choice of ITC Techniques

A comparison of the methods for the principal ITC techniques identified in this SLR is presented in Table 3.

**Figure 4:**
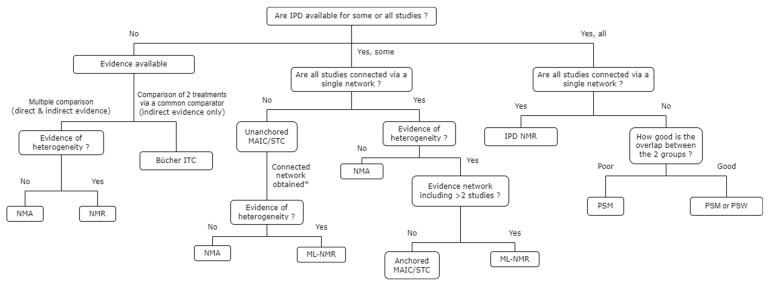
Algorithm for choice of ITC technique. * Unanchored MAIC/STC can be conducted for several pairwise comparisons of interest in order to connect the networks into a single one. Abbreviations: IPD, individual patient data; ITC, indirect treatment comparison; MAIC, matching-adjusted indirect comparison; ML-NMR, multi-level network meta-regression; NMA, network meta-analysis; NMR, network meta-regression; PSM, propensity score matching; PSW, propensity score weighting; STC, simulated treatment comparison.

The availability of the literature that supports decisions around the most appropriate choice of ITC techniques is poor. The findings of this SLR were used to develop an algorithm to help to define the best approach when conducting a feasibility assessment of an ITC [10,54,55]. The choice of ITC technique is critical and should be based on the feasibility of a connected network, the evidence of heterogeneity between and within studies (that arise with the presence of unbalanced TEMs), the overall number of relevant studies and the availability of IPD. The feasibility assessment then informs the type of ITC technique that is feasible. Based on the evidence collected, a proposed decision making process for determining the most appropriate methods to use when conducting an ITC is summarized in Figure 4.

## 4. Discussion

A number of different ITC techniques were identified in the literature, each supported by differing methodologies. An SLR must typically be conducted as the first step for any ITC in order to identify all available studies of interest. The robustness of any ITC depends on the studies upon which it is based, and as such, it is important to assess clinical heterogeneity, consistency and the validity of any included studies.

The technique proposed by Bucher et al. (1997) [16] was the first to ever consider an indirect comparison between two treatments. This approach consists of comparing clinical efficacy or safety estimates of two treatments against a common comparator and combining them to obtain an indirect estimate of the comparison between the two treatments of interest. The introduction of NMAs then extended this concept to a larger network, allowing for the inclusion of as many studies and treatments as needed, as long as the network remains connected. Today, NMAs are a commonly used approach for ITCs, and they were described in 79.5% of articles included in this SLR.

Other population-adjusted methods for ITCs were subsequently developed to overcome issues such as heterogeneity across studies, something that is often encountered when comparing multiple treatments via multiple trials. NMRs adopt a similar methodology to NMAs while also adjusting for heterogeneity between trials by incorporating covariates into a regression model. This method can be conducted using IPD for none, some (ML-NMR), or all (IPD-NMR) trials, depending on availability. MAICs and STCs are also population-adjusted methods, designed for pairwise comparisons only, which can be of particular use in situations where heterogeneity exists across the trials within the network, as well as for incomplete evidence networks. However, the main limitation of these two techniques is the shift of the IPD towards the AgD of the comparator trial, which, thus, defines the target population. MAICs (the second most frequent technique described in this SLR, in 30.1% of articles) and STCs are appropriate techniques for single-arm studies, which are increasingly being conducted in oncology, with a growing number of oncology drug candidates obtaining regulatory approval based on non-comparative trials.

Finally, PS methods such as PSM and IPTW aim to transform two populations that have different characteristics at baseline to increase their similarity and compare them after reducing differences in TEMs. In other words, these techniques are used to improve the balance of potential confounders between two treatments.

This review comprehensively assesses the methods of different ITC techniques described in the literature. A newly published review brings complementary insights on the methodological approaches of identifying TEMs in ITCs. Freitag et al. (2023) found that current ITC guidance mainly focused on developing analytical methods to adjust for TEMs; however, the authors highlighted that there is an urgent need for detailed guidance on the TEM selection process through systematic reviews, formal expert elicitation, and a quantitative assessment of the TEM distribution [56]. ITC methods continue to evolve quickly, and more efficient or more robust techniques may be available in the future. Continuous research to compare the performance of the different methods is, therefore, needed to maintain an understanding of preferred method(s) for ITCs and the optimal use of these in different situations with regard to their robustness.

Some simulation studies have compared the performances of different ITC techniques. For example, the Bucher method has been compared to other ITC techniques to evaluate whether both approaches produce mutually consistent results when used to conduct a given treatment comparison. When compared across different network types, O’Regan et al. (2009) found that NMA and the Bucher method generally produced similar results, particularly in cases where all studies in a network shared a common comparator. Furthermore, a comparison of the Bucher method with meta-regression and logistic regression was conducted by Glenny et al. (2005), specifically in strokes, who concluded that the methods resulted in similar effect estimates and CIs [57,58]; Phillippo et al. (2020) assessed the performances of ML-NMRs, STCs and MAICs and concluded that ML-NMR offers additional advantages over MAIC and STC, particularly as this method extends to larger treatment networks and produces estimates in any target population, making this an attractive choice in a variety of scenarios [46]. Remiro-Azócar et al. (2021) compared MAIC, STC and the Bucher methods for survival outcomes and expressed a preference for MAIC over STC, which produces systematic bias as a result of the non-collapsibility of the log HR. ML-NMR targets a conditional treatment effect but directly avoids the compatibility issues associated with STC. ML-NMR is also applicable in treatment networks of any size, with the two study scenarios as a special case, and could further be adapted to target a marginal treatment effect [14]. Nonetheless, future simulation studies should evaluate population adjustment methods with different outcome types and for instances where assumptions fail.

The use of ITCs has increased rapidly in recent years, and evidence from ITCs is now considered by HTA agencies on a case by case basis. Despite this, their acceptability remains low. Beyond the existing methods guidance issued among HTA agencies in France, England, Germany, Italy, Spain, Australia, and Canada, there is a need for improved methodological advice from HTA agencies in order to guide the development of ITCs for specific scenarios [2,9,10,11,13,17,33]. Moreover, there exists a gap in terms of an international consensus for an ITC methodology that could improve the quality of ITCs submitted to HTA agencies and the rate of ITC acceptance. Similarly, there is an unmet need for guidance on transparent and uniform ways to assess the quality of ITCs submitted to HTA agencies that could help to inform decision making; guidance on the interpretation of ITCs would assist HTA agencies, payers, policymakers and healthcare professionals in using their findings to inform decision making. This is particularly true in light of the EU regulation on HTA, which will apply from 2025; the regulation has the potential to increase consistency in the use and quality of ITCs used to inform HTA across Europe [59].

Although prospective RCTs remain the gold standard mechanism enabling the direct comparison of different therapies, in cases where there is no head-to-head comparison from an RCT, it is imperative to utilize other means of generating comparative data that are meaningful for HTA agencies, payers, clinicians and patients, which can inform complex decision making processes. In this context, ITCs are a well-accepted approach as long as they are robustly implemented, using the optimal methodological approach to fit the context, and transparently discuss the uncertainties and limitations arising from the analysis. However, further guidance to ensure the appropriate choice and techniques for ITC methods will be valuable for optimizing their acceptability moving forward.

### Study Limitations

Overall, the comparison of the performance of the ITC techniques identified within this SLR is limited due to the scarcity of such studies. Among the little evidence available, the comparisons are limited to a specific context (for example, one treatment, one population, one endpoint and the required assumptions). However, the objective of this SLR was not to provide a detailed comparative analysis of the performance of ITC techniques, and, therefore, further research is still needed to do so [57,58].

This review has a number of further limitations. Firstly, a standard systematic search of electronic databases could not be carried out due to the searches returning an unmanageable number of hits, and an alternative approach was, therefore, required. However, this approach was specifically designed to thoroughly identify all relevant articles of interest to meet the objective of the review. Furthermore, the scope of this SLR was to focus on articles discussing ITC techniques and their methodology; since there is no SLR checklist for such articles, a standard quality assessment of the articles was not possible. However, the inclusion of only peer-reviewed publications complemented by publications from HTA and regulatory agencies ensured the quality of the review. The evidence included in the review also has its own limitations; in the literature, there is a lack of consensus on the terminology associated with ITCs, and the assumptions upon which indirect comparisons are based are often not clearly defined.

## 5. Conclusions

ITCs provide robust evidence where direct comparative evidence is unavailable and, as such, can provide a useful source of evidence for informing decision making in medicine. Although ITCs are now considered by HTA agencies on a case by case basis, their acceptability remains low, and there is a need for further clarity regarding the use of appropriate techniques and the assessment of their results in order to improve the quality of ITCs submitted to HTA agencies. ITC methods continue to evolve quickly, and more efficient techniques may be available in the future. Continuous research is, therefore, needed to compare the performances of different ITC methods and the robustness of their applicability in specific contexts.

## Figures and Tables

**Table 1:** Additional search inclusion and exclusion criteria.

Inclusion criteria
Studies reporting the methodology for ITC techniques and/or the advantages/limitations of ITC techniques Recommendations/guidelines/guidance/good practice publications on ITC methodologies Illustration/application studies with a focus on methodologies Simulation studies (which compare the performance(s) of one or more ITC techniques under different assumptions/data structures or using different methods) Articles presenting general methods of further statistical considerations for ITC techniques, including but not limited to frequentist versus Bayesian approach, fixed-effect and random-effect models, ranking methodology, assessments of heterogeneity and/or inconsistency
Exclusion criteria
Application studies without a methodological focus Studies/guidance on the reporting of ITC techniques only Practical frameworks to assess the methodological robustness and reliability of results from ITCs Surveys discussing the use of ITCs Articles on the application of ITCs to inform the design of future trials Tools/software to perform ITCs, for example programming, visualization, graphical display, automated generation of Bayesian models

Abbreviations: ITC, indirect treatment comparison.

**Table 2:** Terminology and definitions.

Assumptions	
Homogeneity	No variation in the treatment effect between trials within a pairwise comparison, i.e., for each pairwise comparison, the relative efficacy of each treatment is the same across all trials. This is induced by the similarity of trials (in terms of study design, patient characteristics, treatments and outcomes measured) concerning the relevant treatment effect for each pairwise comparison.
Similarity or transitivity	Similarity of all the trials that contribute to an ITC in terms of study design, patient characteristics, treatments, and outcomes measured. This relies on the similarity of trials with regard to TEMs that may impact the relevant treatment effect between pairwise comparisons that contribute to an ITC.
Consistency	No variation in the treatment effect between pairwise comparisons, therefore leading to the same treatment effect produced by direct and indirect estimates. Consistency is equal to transitivity across a simple triangular loop.
Exchangeability	Combination of similarity, homogeneity and consistency assumptions.
Connectivity	Existence of common comparators to connect the network.
Constancy of treatment effect	Treatment effects are constant across trial populations: constancy of relative effects (NMA); conditional constancy of relative effects (anchored population-adjusted indirect comparison); conditional constancy of absolute effects (unanchored population-adjusted indirect comparison).
**Other definitions**	
Treatment effect modifier (TEM)	Patient or study characteristic that influences the treatment effect on a clinical outcome (impacts the relative treatment effect).
Prognostic factor	Patient or study characteristic that influences clinical outcomes, regardless of the intervention and comparator (impacts the absolute treatment effect).

Abbreviations: ITC, indirect treatment comparison; NMA, network meta-analysis; TEM, treatment effect modifier.

**Table 3:** Summary of the methods for the ITC techniques identified in the SLR.

ITC Methods	Standard Techniques		Population-Adjusted Techniques				
	Bucher ITC	NMA	MAIC	STC	NMR	PSM	IPTW
Number of treatments compared	2	**Unlimited**	2	2	**Unlimited**	2	2
Need for IPD	No	No	Yes, for at least one trial	Yes, for at least one trial	No for NMR Yes for ML-NMR	Yes for all trials	Yes for all trials
Possible inclusion of single-arm trials	No	No	**Yes**	**Yes**	No	**Yes**	**Yes**
Requires a connected network	Yes	Yes	**No**	**No**	Yes	**No**	**No**
Allows random- and fixed-effect approaches	NA	**Yes**	NA	NA	**Yes**	NA	NA
Allows the inclusion of any type of outcomes	**Yes**	**Yes**	**Yes**	**Yes**	No for TTE	**Yes**	Yes
**Assumptions required**							
Homogeneity	Yes	Yes	Yes	Yes	Yes ^a^	Yes	Yes
Similarity	Yes	Yes	No	No	No	No	No
Consistency	NA	Yes	No	No	Yes	NA	NA
Constancy of TE ^b^	Yes	Yes	Yes	Yes	Yes	No	No
Other	Independence between pairwise comparisons	/	No unobserved prognostic factors or TEM	No unobserved prognostic factors or TEM	No unobserved prognostic factors or TEM	No unobserved prognostic factors or TEM	No unobserved prognostic factors or TEM
**Strengths**	/	Unlimited number of trials	Adjust for TEM No need for a common comparator		Applicable to a network of any size Assess and adjust heterogeneity and TEM	Adjust for confounders introducing heterogeneity Patients can be excluded if too different	Adjust for confounders introducing heterogeneity Preserves sample size
**Limitations**	Limited to simple networks (3 treatments) and 2-arm trials Does not adjust for TEM	Does not adjust for TEM	Limited to pairwise comparisons Biased estimates if unobserved TEM		Rarely feasible, as it requires an important number of trials	Biased estimates if unobserved prognostic factors and TEM	

^a^ Not for ML-NMR. ^b^ For NMA and NMR: constancy of relative effects; for anchored MAIC and STC and ML-NMR: conditional constancy of relative effects; for unanchored MAIC and STC: conditional constancy of absolute effects. Note: The characteristic of a technique is highlighted in green when it is an advantage. Abbreviations: IPD, individual patient data; IPTW, inverse-probability treatment weighting; ITC, indirect treatment comparison; MAIC, matching-adjusted indirect comparison; ML-NMR, multi-level network meta-regression; NA, not applicable; NMA, network meta-analysis; NMR, network meta-regression; PSM, propensity score matching; SLR, systematic literature review; STC, simulated treatment comparison; TE, treatment effect; TEM, treatment effect modifier; TTE, time-to-event outcome.

**Table 4:** Summary of main differences between frequentist and Bayesian frameworks.

	Frequentist	Bayesian
Probability	Probability of the data given a hypothesis (likelihood) 95% CI gives estimates of how many times, out of 100 trials, the point estimate will be found	Conditional probabilities: probability of a hypothesis given the data and the prior distribution of the parameter 95% CrI gives the probability that the point estimate lies within the interval
Uncertainty	Unknown parameters are assumed to be fixed, and data are repeatedly taken from random samples	Unknown parameters are treated probabilistically and estimated based on simulations
Prior information	None	Prior distributions are used to estimate treatment effect, and possibly between-study heterogeneity, so as not to influence the results (results driven by the data only)
Interpretation	Point estimate and dispersion (CI) around it	Ranking, probabilities of being best, second best, etc.

Abbreviations: CI, confidence interval; CrI, credibility interval.

## Supplementary Materials

The following supporting information can be downloaded via this link: https://www.mdpi.com/article/10.3390/jmahp12020006/s1, Table S1 Search via Embase.com (15 November 2021); Table S2 PubMed search via https://pubmed.ncbi.nlm.nih.gov/ (15 November 2021); Table S3 List of pre-defined papers; Table S4 PICO eligibility criteria.
