# Supplementary material for: Methods for Indirect Treatment Comparison: Results from a Systematic Literature Review
Source: J Mark Access Health Policy. 2024 Apr 16;12(2):58–80. doi: 10.3390/jmahp12020006 (PMC11036291; doi:10.3390/jmahp12020006)
Supplement: Supplementary file 1 [file jmahp-12-00006-s001.zip › jmahp-2713799-supplementary.pdf]

Table S1. PICO eligibility criteria.

Criteria	Include	Exclude
Population	-	Animals/in vitro data
Intervention / Comparator	No restrictions, any comparator will be included	-
Outcomes	No restrictions, any outcome will be included	-
Study design / setting	• Methodology papers<br>• Studies<br>• Reviews	• Animal / in-vitro studies<br>• PK/PD studies<br>• Cost studies (cost-effectiveness, budget impact analyses)<br>• Studies describing the application of ITC methods in the context of specific treatments
Language of publication	English language publications	Non-English language publications without an English abstract
Date of publication	• Full publications: from 1997<br>• Hand-searched: Not restricted	-
Countries	No restriction	-

Abbreviations: ITC: indirect treatment comparison; PD: pharmacodynamic; PICO: patient/population, intervention, comparison, outcome; PK: pharmacokinetic..

Table S2. Search via Embase.com (15 November 2021).

Concept	Search no.	Query	Results
Journal	#1	'british medical journal':jt	96,300
Journal	#2	'the bmj':jt	4,340
Journal	#3	'statistics in medicine':jt	10,061
Journal	#4	'journal of the royal statistical society':jt	50
Journal	#5	'journal of the royal statistical society, series a' OR 'journal of the royal statistical society: series a'	44
Journal	#6	'journal of the royal statistical society, series b' OR 'journal of the royal statistical society: series b'	69
Journal	#7	'j. r. stat. soc.' OR 'j r stat soc'	96
Journal	#8	'journal of comparative effectiveness research':jt	917
Journal	#9	'international journal of technology assessment in health care':jt	3,352
Journal	#10	'pharmacoeconomics':jt	5,937
Journal	#11	'journal of clinical epidemiology':jt	7,119
Journal	#12	'statistical methods in medical research':jt	2,099
Journal	#13	'medical decision making':jt	2,759
Journal	#14	'journal of the american statistical association':jt	124
Journal	#15	'j. amer. statist. assoc.' OR 'j amer statist assoc' OR 'j. am. stat. assoc.' OR 'j am stat assoc'	202
Journal	#16	'bmc medical research methodology':jt	2,647
Journal	#17	'value in health':jt	50,035
Journal	#18	'journal of clinical epidemiology':jt	7,119
Journal	#19	'journal of biopharmaceutical statistics':jt	1,884
Journal	#20	'biostatistics':jt	1,766
Journal	#21	'pharmaceutical statistics':jt	1,038
Journal	#22	'research synthesis methods':jt	384

Journals – combined search	#23	#1 OR #2 OR #3 OR #4 OR #5 OR #6 OR #7 OR #8 OR #9 OR #10 OR #11 OR #12 OR #13 OR #14 OR #15 OR #16 OR #17 OR #18 OR #19 OR #20 OR #21 OR #22	190,941
Author	#24	'abrams k':au	608
Author	#25	'ades a':au	364
Author	#26	'bucher h':au	967
Author	#27	'bujkiewicz s':au	53
Author	#28	'caldwell d':au	750
Author	#29	'dias s':au	1,109
Author	#30	'efthimiou o':au	65
Author	#31	'higgins j':au	2,710
Author	#32	'phillippo d':au	12
Author	#33	'salanti g':au	232
Author	#34	'signorovitch j':au	380
Author	#35	'song f':au	3,268
Author	#36	'sutton a':au	1,207
Author	#37	'swallow e':au	174
Author	#38	'welton n':au	236
Authors – combined	#39	#24 OR #25 OR #26 OR #27 OR #28 OR #29 OR #30 OR #31 OR #32 OR #33 OR #34 OR #35 OR #36 OR #37 OR #38	11,590
Network meta-analysis	#40	'network meta-analysis'/exp	4,765
Indirect treatment comparison/meta-analysis	#41	(('meta analysis'/exp OR 'meta analys$s' OR 'meta-analys$s') AND (indirect OR indirectly OR 'cross-stud*' OR 'cross-trial$' OR 'multiple treatment$' OR 'mixed treatment$' OR 'multiple comparison$'))	7,053
Indirect treatment comparison/meta-analysis	#42	'indirect treatment comparison$' OR 'indirect-treatment-comparison$' OR 'adjusted indirect comparison$' OR 'nondirect comparison$' OR 'non-direct comparison$' OR 'maic' OR 'matching adjusted indirect comparison$' OR 'matching-adjusted indirect comparison$' OR 'matched adjusted indirect comparison$' OR 'network metaanalys$s' OR 'network meta-analys$s' OR 'indirect comparison$' OR (indirect NEAR/2 compar*) OR 'itc' OR 'nma' OR 'network meta-regression$' OR 'ml-nmr'	27,251
Indirect treatment comparison/meta-analysis/network meta-analysis/network meta-regression	#43	#40 OR #41 OR #42	30,614
Authors or journals of interest	#44	#23 OR #39	201,886
Authors or journals of interest, and on methodology of interest	#45	#43 AND #44	2,788
Limit to studies published from 1997 onwards	#46	#45 AND [1997-2021]/py	2,782
Limit to published articles, articles in press and reviews published from 1997 onwards	#47	#46 AND (‘article’/it OR ‘article in press’/it OR ‘review’/it)	**897**

Table S3. PubMed search via https://pubmed.ncbi.nlm.nih.gov/ (15 November 2021).

Concept	Search no.	Query	Results
Journal	#1	british medical journal [ta]	195,866
Journal	#2	"the bmj"	4,544
Journal	#3	statistics in medicine [ta]	9,634
Journal	#4	journal of the royal statistical society [ta]	255
Journal	#5	"journal of the royal statistical society, series a" OR "journal of the royal statistical society: series a"	8
Journal	#6	"journal of the royal statistical society, series b" OR "journal of the royal statistical society: series b"	155
Journal	#7	"j. r. stat. soc." OR "j r stat soc"	416
Journal	#8	journal of comparative effectiveness research [ta]	920
Journal	#9	international journal of technology assessment in health care [ta]	2,637
Journal	#10	pharmacoeconomics [ta]	3,405
Journal	#11	journal of clinical epidemiology [ta]	6,923
Journal	#12	statistical methods in medical research [ta]	1,996
Journal	#13	medical decision making [ta]	2,688
Journal	#14	journal of the american statistical association [ta]	738
Journal	#15	"j. amer. statist. assoc." OR "j amer statist assoc" OR "j. am. stat. assoc." OR "j am stat assoc"	798
Journal	#16	bmc medical research methodology [ta]	2,606
Journal	#17	value in health [ta]	5,433
Journal	#18	journal of biopharmaceutical statistics [ta]	1,837
Journal	#19	biostatistics [ta]	1,223
Journal	#20	pharmaceutical statistics [ta]	858
Journal	#21	research synthesis methods [ta]	493
Journals – combined search	#22	#1 OR #2 OR #3 OR #4 OR #5 OR #6 OR #7 OR #8 OR #9 OR #10 OR #11 OR #12 OR #13 OR #14 OR #15 OR #16 OR #17 OR #18 OR #19 OR #20 OR #21	237,984
Author	#23	abrams k [au]	433
Author	#24	ades a [au]	324
Author	#25	bucher h [au]	627
Author	#26	bujkiewicz s [au]	33
Author	#27	caldwell d [au]	755
Author	#28	dias s [au]	754
Author	#29	efthimiou o [au]	59
Author	#30	higgins j [au]	2,164
Author	#31	phillippo d [au]	14
Author	#32	salanti g [au]	214
Author	#33	signorovitch j [au]	143
Author	#34	song f [au]	2,949
Author	#35	sutton a [au]	1,047
Author	#36	swallow e [au]	126
Author	#37	welton n [au]	199
Authors – combined	#38	#23 OR #24 OR #25 OR #26 OR #27 OR #28 OR #29 OR #30 OR #31 OR #32 OR #33 OR #34 OR #35 OR #36 OR #37	9,378
Network meta-analysis	#39	network meta-analysis [MeSH Terms]	2,992
Indirect treatment comparison/meta-analysis	#40	(("meta analysis as topic"[MeSH Terms] OR "meta analysis" OR "meta analyses" OR "meta-analysis" OR "meta-analyses" OR	4,464

		metaanalysis OR metaanalyses) AND (indirect OR indirectly OR cross-stud* OR cross-trial OR "multiple treatment" OR "mixed treatment" OR "multiple comparison" OR indirect-comparison))	
Indirect treatment comparison/meta-analysis	#41	"indirect treatment comparison*" OR "indirect-treatment-comparison*" OR "adjusted indirect comparison*" OR "nondirect comparison*" OR "non-direct comparison*" OR maic OR "matching adjusted indirect comparison*" OR "matching-adjusted indirect comparison*" OR "network meta analysis" OR "network meta analyses" OR "matched adjusted indirect comparison*" OR "network metaanalys*" OR "network meta-analys*" OR "network meta analysis" OR "indirect comparison*" OR (indirect AND compar*) OR itc OR itcs OR nma OR "network meta-regression*" OR "network meta regression*" OR "network metaregression*" OR ml-nmr	83,320
Indirect treatment comparison/meta-analysis/network meta-analysis/network meta-regression	#42	#39 OR #40 OR #41	84,828
Authors or journals of interest	#43	#22 OR #38	246,826
Authors or journals of interest, and on methodology of interest	#44	#42 AND #43	1,305
Limit to studies published from 1997 onwards	#45	#44 AND 1997/01/01:2021/12/12[dp]	1,239
Limit to published journal articles, published from 1997 onwards	#46	#44 AND 1997/01/01:2021/12/12[dp] Filters: Journal article	1,200
Limit to reviews published from 1997 onwards	#47	#44 AND 1997/01/01:2021/12/12[dp] Filters: Review	292
Limit to technical reports published from 1997 onwards	#48	#44 AND 1997/01/01:2021/12/12[dp] Filters: Technical report	1
Limit to published journal articles, technical reports, and reviews published from 1997 onwards	#49	#46 OR #47 OR #48	1,200
Limit to studies published from 1997 onwards, added to PubMed in scope of MEDLINE, for which MeSH terms are not yet assigned	#50	(#44 AND 1997/01/01:2021/12/12[dp]) AND inprocess[sb]	18
Limit to reviews, technical reports, journal articles, PUBMED-IN-PROCESS articles published from 1997 onwards	#51	#49 OR #50	1,201

Footnote: Combining the PubMed and Embase searches above, after de-duplication yields 1,397 records.

Table S4. List of pre-defined papers.

No.	Reference	Identified in Search?	ID no. (EndNote* or hand search)
1	Welton, N. J. (2020). CHTE2020 SOURCES AND SYNTHESIS OF EVIDENCE; UPDATE TO EVIDENCE SYNTHESIS METHODS. CHTE2020 sources and synthesis of evidence | NICE Decision Support Unit | The University of Sheffield	Y	Hand search
2	Bucher HC, Guyatt GH, Griffith LE, Walter SD. The results of direct and indirect treatment comparisons in meta-analysis of randomized controlled trials. J Clin Epidemiol 1997; 50(6): 683-691.	Y	38
3	Bujkiewicz, S., Achana, F., Papanikos, T., Riley, R., & Abrams, K. (2019). NICE DSU Technical Support Document 20. Multivariate meta-analysis of summary data for combining treatment effects on correlated outcomes and evaluating surrogate endpoints. Full list of technical support documents (TSDs) | NICE Decision Support Unit | The University of Sheffield	Y	Hand search
4	Dias, S., Welton, N.J., Sutton, A.J. & Ades, A.E. (2011). NICE DSU Technical Support Document 2. A General Linear Modelling Framework for Pairwise and Network Meta-Analysis of Randomised Controlled Trials. Full list of technical support documents (TSDs) | NICE Decision Support Unit | The University of Sheffield	Y	155
5	Dias, S., Sutton, A. J., Welton, N. J., & Ades, A. E. (2016). NICE DSU Technical Support Document 3. Heterogeneity: subgroups, meta-regression, bias and bias-adjustment<br>Full list of technical support documents (TSDs) | NICE Decision Support Unit | The University of Sheffield	Y	137
6	Efthimiou, O., Mavridis, D., Debray, T. P., Samara, M., Belger, M., Siontis, G. C., ... & GetReal Work Package 4. (2017). Combining randomized and non-randomized evidence in network meta-analysis. *Statistics in medicine*, *36*(8), 1210-1226.	Y	345
7	Higgins, J. P., S. G. Thompson, et al. (2003). "Measuring inconsistency in meta-analyses." BMJ 327(7414): 557-560. https://www.ncbi.nlm.nih.gov/pmc/articles/PMC192859/pdf/3270557.pdf	N	-
8	Phillippo, D., Ades, T., Dias, S., Palmer, S., Abrams, K. R., & Welton, N. (2016). NICE DSU Technical Support Document 18: methods for population-adjusted indirect comparisons in submissions to NICE. Full list of technical support documents (TSDs) | NICE Decision Support Unit | The University of Sheffield	Y	28
9	Phillippo, D. M., Dias, S., Elsada, A., Ades, A. E., & Welton, N. J. (2019). Population adjustment methods for indirect comparisons: a review of national institute for health and care excellence technology appraisals. *International journal of technology assessment in health care*, *35*(3), 221-228. https://www.ncbi.nlm.nih.gov/pmc/articles/PMC6650293/pdf/EMS83347.pdf	Y	69
10	Phillippo, D. M., Dias, S., Ades, A. E., Belger, M., Brnabic, A., Schacht, A., ... & Welton, N. J. (2020). Multilevel network meta-regression for population-adjusted treatment comparisons. *Journal of the Royal Statistical Society: Series A (Statistics in Society)*, *183*(3), 1189-1210. https://www.ncbi.nlm.nih.gov/pmc/articles/PMC7362893/pdf/RSSA-183-1189.pdf	Y	946

11	Sutton, A., Ades, A. E., Cooper, N., & Abrams, K. (2008). Use of indirect and mixed treatment comparisons for technology assessment. *Pharmacoeconomics*, *26*(9), 753-767.	Y	254
12	Swallow, E., Patterson-Lomba, O., Ayyagari, R., Pelletier, C., Mehta, R., & Signorovitch, J. (2020). Causal inference and adjustment for reference-arm risk in indirect treatment comparison meta-analysis. *Journal of Comparative Effectiveness Research*, *9*(10), 737-750. https://pubmed.ncbi.nlm.nih.gov/32490682/	Y	60

*EndNote database of 1,397 references retrieved from PubMed and Embase.com searches, following de-duplication.
